# THz image recognition of moldy wheat based on multi-scale context and feature pyramid

**DOI:** 10.3389/fpls.2025.1490384

**Published:** 2025-06-04

**Authors:** Yuying Jiang, Xinyu Chen, Hongyi Ge, Xixi Wen, Mengdie Jiang, Yuan Zhang

**Affiliations:** ^1^ Key Laboratory of Grain Information Processing & Control, Ministry of Education, Henan University of Technology, Zhengzhou, China; ^2^ Henan Provincial Key Laboratory of Grain Photoelectric Detection and Control, Zhengzhou, China; ^3^ School of Artificial Intelligence and Big Data, Henan University of Technology, Zhengzhou, China; ^4^ College of Information Science and Engineering, Henan University of Technology, Zhengzhou, China

**Keywords:** terahertz, identification of moldy wheat, spectral image, image classification, deep learning

## Abstract

Wheat is susceptible to mold growth due to storage conditions, which subsequently affects its quality; therefore, timely and rapid identification of moldy wheat is critically important. In order to achieve high-precision recognition and class classification of wheat with different degrees of mold, a multi-scale context and feature pyramid based moldy wheat recognition network (MSCFP-Net) is proposed. Firstly, the network uses the residual network ResNeXt as the baseline network, and incorporates a multi-scale contextual feature extraction module, which is more helpful to determine the important discriminative regions in the whole image to extract more image detail features. In addition, a coordinated attention mechanism module is introduced to perform global average pooling from both directions to learn the importance of different regions in the input features in a dynamically weighted manner. Moreover, a bidirectional feature pyramid network is embedded into the baseline model, so that certain coarse-grained features and fine-grained features are retained in the processed output features at the same time to improve the network recognition accuracy. Compared with the baseline network, the four evaluation indexes of Accuracy, Precision, Recall and F1-Score of MSCFP-Net are improved by 1.08%, 1.25%, 0.53% and 0.91%, respectively. In addition, a series of comparison experiments and ablation experiments show that the classification network constructed in this paper has the best fine-grained classification performance for moldy wheat THz images.

## 1 Introduction

Wheat is rich in nutrients, widely grown, and is one of the important reserve grains in China. However, in the storage process, its internal sugars, fats, proteins and other nutrients are very easy to be decomposed by microorganisms to produce mold, resulting in the phenomenon of mold. In order to prevent more stored wheat from being contaminated and further affecting the overall quality, timely and rapid screening of moldy wheat is important for the safety of stored grain. Traditional screening means are mainly manual sensory evaluation, biochemical method, electronic nose detection, etc., which have shortcomings such as strong subjectivity, low precision, high requirements for professionalism in operation, and contamination and damage of test samples.

Terahertz (THz) radiation (Wan et al., 2020), also known as THz wave, is electromagnetic radiation with a frequency range between 0.1 and 10 THz, which is located in the transition region between macroscopic electronics and microscopic photonics. Since the rotational and vibrational jumps between or within many molecules, the backbone vibrations of nucleic acid macromolecules, and the low-frequency vibrational absorption frequencies of the lattice in crystals are all in the THz band range, the THz technique is well suited for the detection of bio-macromolecular substances. THz wave has the technical characteristics of non-destructive penetration and low photon energy, which can not only get the surface information of the sample, but also obtain the detailed information of the internal structure of the sample and other details to achieve non-destructive detection. With the development of novel metamaterials and tunable devices, THz devices have made significant progress in detection sensitivity and tunable frequency bands. Zeng et al. (2025) proposed a square circular Dirac semimetal terahertz tunable absorber, incorporating new materials, and developed a frequency tunable, simple structured, and easy to manufacture vibration absorber. Li et al. (2025) designed a tunable broadband metamaterial absorber based on graphene, achieving an ultra-wide absorption bandwidth from 3.8 - 7.19 THz with an absorption rate exceeding 90%. The design and optimization of THz metamaterial devices provide a stronger hardware foundation for the application of THz technology. At present, THz technology have achieved significant breakthrough in many fields, such as agricultural product safety detection (Ge et al., 2024; Tu et al., 2025), biomedicine (Koul and Kaurav, 2022; Zamzam et al., 2025) and security inspection (Zeng et al., 2024).

The unique nature of THz wave makes it of great potential application value in the field of agricultural product quality inspection. In recent years, THz technology has been applied in this field and obtained corresponding results, such as identification of genetically modified crops, food adulteration, moisture content and so on. Liu et al. (2023) used spectra in the 0.2-1.5 THz band to collect the data of pericarpium citri reticulatae and constructed a CNN recognition model to identify the samples from different years, with a recognition accuracy of 95.63%. Wei et al. (2020) collected THz time-domain spectra of 225 transgenic and non-transgenic soybean samples, took out the interfering spectral bands by interval partial least squares (iPLS), and identified the transgenic samples by using grid search- support vector machine (Grid Search-SVM), with an identification accuracy of 96.15%. Chen et al. (2020) combined the collected THz spectral data with chemometric methods to detect the extent of pesticide residues, and the discrimination coefficients obtained were all greater than 0.91 based on the absorption peaks of the three pesticides in the 0.4-1.7 THz region. The above studies used preprocessing operations such as dimensionality reduction through THz spectral technique, combined with a series of machine learning-based algorithms such as PCA, for sample THz spectral data, and analyzed them qualitatively or quantitatively with models such as CNN, SVM and LS-SVM, which all achieved excellent experimental results. Hu et al. (2024) took dried longans as the research object and used THz transmission imaging to collect images of dried longans with different fullness degrees. Firstly, the THz spectral signals of different regions of interest were analyzed to identify the origin of dried longans, and the identification accuracy rate can reach 98.57%. The fullness of longan was calculated by combining the method of Otsu threshold segmentation and image inversion, and the maximum error was less than 3.11%. Jiang et al. (2024) applied THz imaging technology to the detection of peanut seed quality. By taking advantage of the characteristic that THz waves can penetrate peanut shells and combining with CNN, the identification of standard, mildewed, defective, dried and germinated peanut seeds was achieved. The recognition accuracy rate reached 98.7%. THz technology can be combined with digital signal processing and spectral analysis to analyze the THz spectral response characteristics of different kinds of agricultural products, and achieve high-precision prediction results through the constructed machine learning and deep learning models.

In this paper, deep learning techniques are introduced so as to achieve high-precision recognition and classification of moldy wheat. In order to fully extract the target feature information of the discriminative region in the moldy wheat image and achieve high-precision fine-grained image classification, this paper constructs a THz image recognition network for moldy wheat based on multi-scale context and feature pyramid, using ResNeXt as the baseline network.

Firstly, a multi-scale contextual feature extraction module is designed and embedded in ResNeXt to obtain features at different scales and improve the image recognition accuracy. Secondly, for the fine-grained image classification task that needs to give more attention to the discriminative features in the target region so as to achieve high-precision classification for samples of the same category with insignificant differences, the coordinated attention mechanism module is introduced. Finally, the bidirectional feature pyramid network is embedded in the baseline network, thus realizing the fusion of deep semantic features and shallow fine-grained features to obtain the output features of multi-scale fusion.

## 2 Experimental materials and methods

### 2.1 Experimental equipment and principles

The THz spectral imaging system used in this experiment is the terahertz 3D chromatographic imaging system (QT-TO1000) of Qingdao Quenda Terahertz Technology Co., Ltd. in China. The THz detection system used in this paper consists of a total of four parts, namely the transmitter and receiver, the optical path system, the sample platform and the mobile system. Firstly, the sample is placed on the two-dimensional scanning platform. In the transmitter module, the pulse signal is generated by the fiber optic femtosecond laser, the photoconductive antenna converts the femtosecond pulse signal into a picosecond THz pulse signal, and a short THz pulse is emitted from the transmitter source, which is focused on the surface of the sample through the optical path system and penetrates into the measured sample. Due to the different refractive indices between the different thicknesses inside a sample, when the THz wave passes through the interfaces of different thicknesses, it will emit part of the reflected wave, which is received by the detector, and the detected THz signal is converted into an electrical signal by the receiver module.

In this paper, reflectance imaging mode was used for the experimental samples. The samples were uniformly placed on a movable two-dimensional scanning platform, and the maximum scanning range of the system is 100 mm×100 mm with a spatial resolution of 0.1 mm. Each pixel point acquires the THz time-domain waveform of 9000 time-domain points within a scanning time range of 90 ps. The acquired image data was stored in three-dimensional form and contains both spatial and spectral information.

### 2.2 Sample preparation and data acquisition

**Figure 1:**
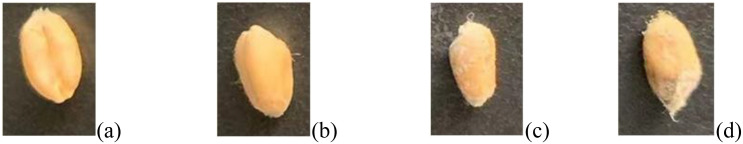
Wheat samples with different degrees of mold: **(a)** Normal. **(b)** Slightly moldy. **(c)** Moderately moldy. **(d)** Seriously moldy.

The wheat variety used in the experiment is No.22 Fine Wheat. According to the moldy wheat culture experiment in the literature (He et al., 2019), normal wheat grains with smooth surface were selected and their moisture was adjusted to 18%, and then placed in a constant temperature and humidity chamber with a preset temperature of 35 °C. Part of the wheat grains were taken out on each of the third, sixth, and ninth days as slightly moldy, moderately moldy, and seriously moldy samples, respectively, so as to obtain the samples with four degrees of mold as shown in **Figure 1**.

As seen in **Figure 1**, wheat with different degrees of mold had complete profile information, but the surfaces had slight differences due to different degrees of mold encapsulation. **Figure 1a** had the smoothest surface with no mold encapsulation. Compared to normal wheat, slightly moldy wheat has slight traces of mold encapsulation on the surface, and seriously moldy wheat has the deepest degree of mold encapsulation and the roughest surface.

**Figure 2:**
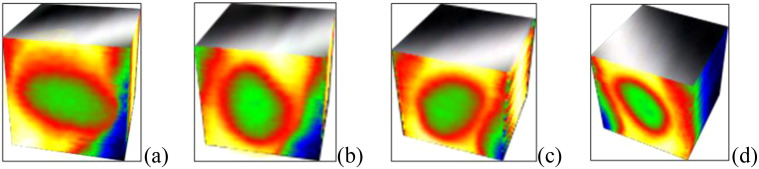
3D THz images of wheat samples with different degrees of mold: **(a)** Normal. **(b)** Slightly moldy. **(c)** Moderately moldy. **(d)** Seriously moldy.

A total of 1200 wheat kernels with different levels of mold were collected in this experiment, including 300 samples each of normal wheat, slightly moldy wheat, moderately moldy wheat, and seriously moldy wheat. The original 3D THz data of the wheat samples were first subjected to Fourier transform, resulting in frequency-domain data with a size of 180×180×300. **Figure 2** demonstrates the 3D THz images of wheat samples obtained by the imaging system. Subsequently, each 3D image was sliced into 300 two-dimensional THz images of moldy wheat with a resolution of 180×180.

**Figure 3:**
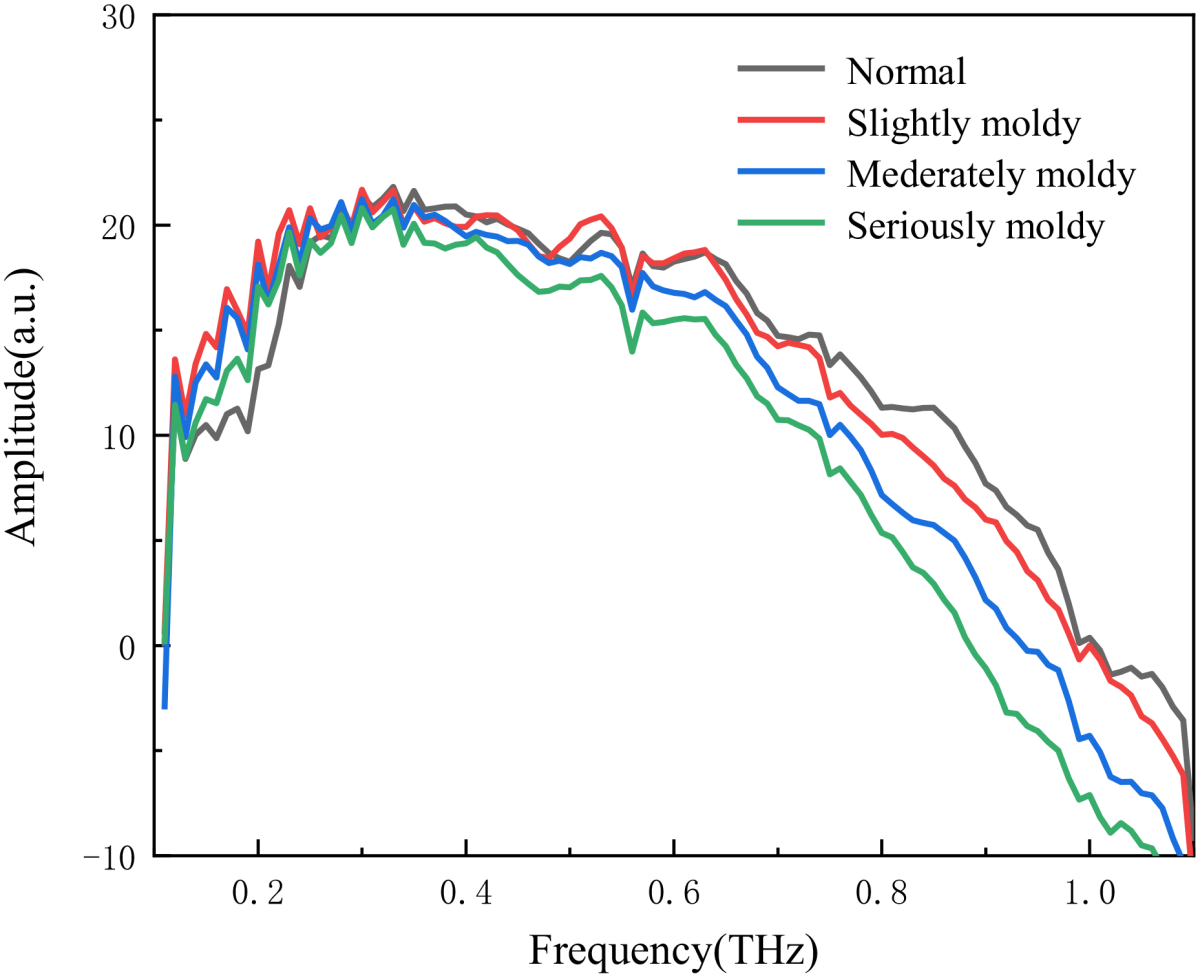
THz frequency domain spectra of four types of samples.

The original signal acquired by the 3D chromatographic imaging system is time-domain signal, and then the frequency-domain signal is obtained by Fourier transform. The frequency domain signal contains the internal structure information of the sample, so we use the original signal converted by the Fourier transform to get the frequency domain signal as the data base. The frequency domain spectra of the four types of samples are shown in **Figure 3**, and the amplitude values of all the samples are the highest near 0.3 THz, and gradually weakened after 0.5 THz.

**Figure 4:**
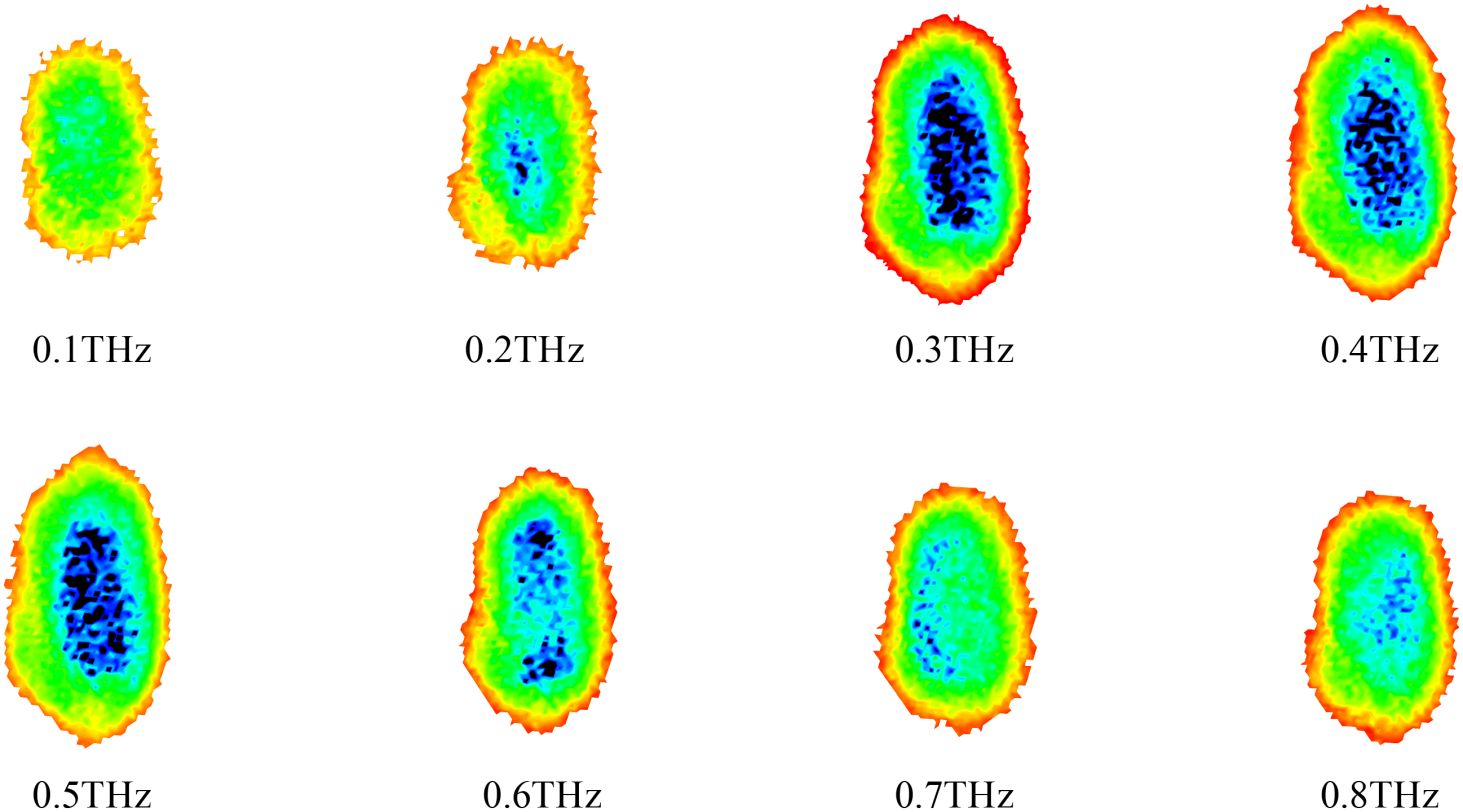
THz images of normal wheat samples at different frequencies.

After slicing the original three-dimensional THz images, a background removal process was applied to retain only the main parts of the wheat samples, resulting in background-free two-dimensional images. Taking normal wheat samples as an example, **Figure 4** shows THz images at different frequencies. As can be observed, at 0.1 and 0.2 THz, the images contain limited usable features and exhibit weak signal intensity, making them unsuitable for effective feature extraction. In contrast, at 0.3 and 0.4 THz, the wheat contours are clearly visible, the signal intensity is relatively strong, and the internal structure of the grains is well preserved. As the frequency further increases to the range of 0.5 - 0.8 THz, both the contour and internal structural information of the wheat samples gradually diminish. Therefore, based on the frequency-domain spectral analysis in **Figure 3**, the 0.3 THz images were selected for use in subsequent experiments. A total of 300 THz images were obtained for each category of wheat with different mold levels, resulting in 1200 images used as the dataset for the model.

**Figure 5:**
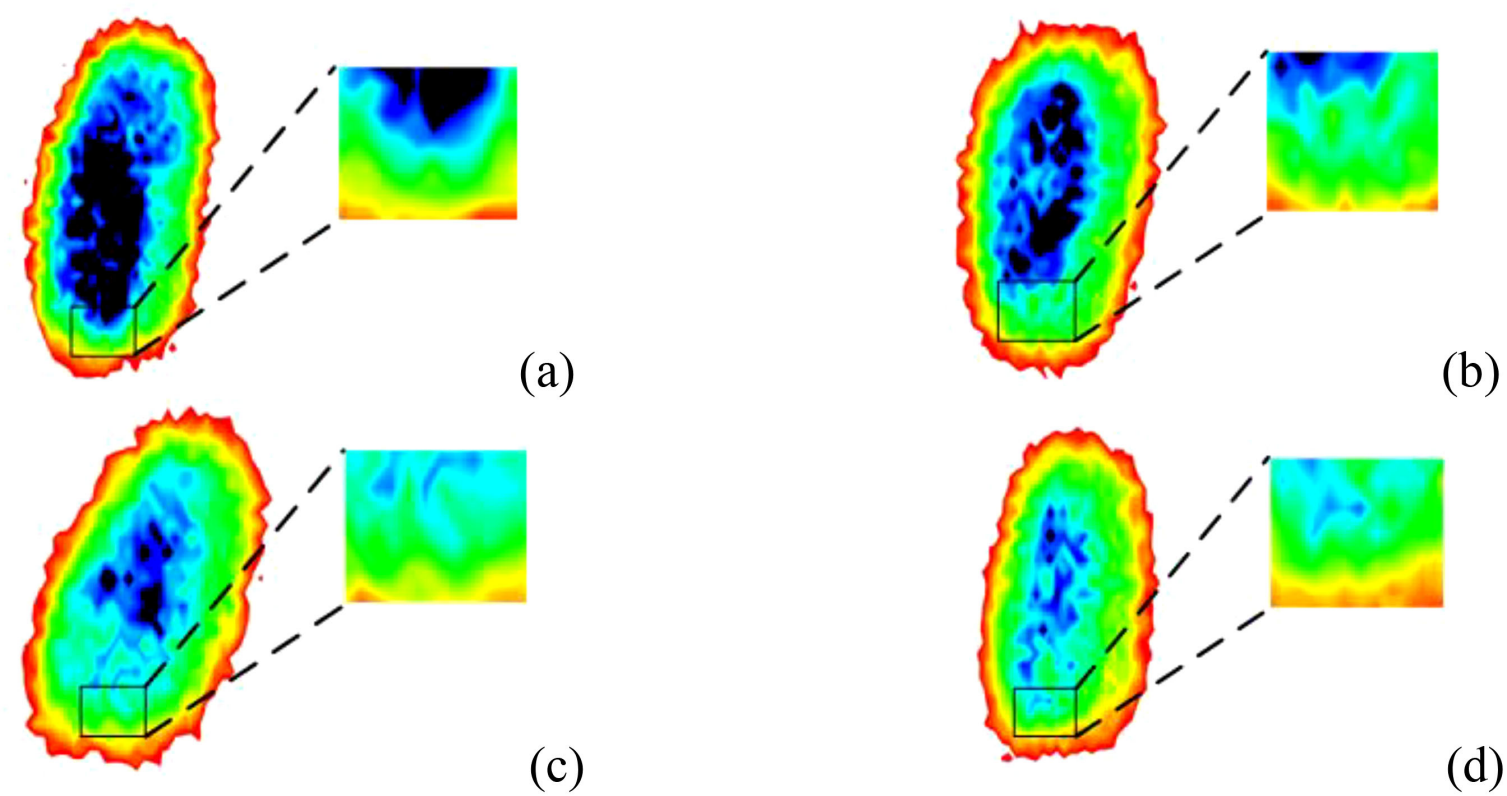
THz images of the samples after removing the background: **(a)** Normal. **(b)** Slightly moldy. **(c)** Moderately moldy. **(d)** Seriously moldy.

As can be seen from **Figure 5**, all four types of sample wheat after removing the background have complete outlines, and the main body of the sample is expressed completely. Among them, the blue area in **Figure 5a** has the largest area and the darkest color, which indicates that its internal structure has the most complete expression and there is no mold wrapping on the surface. The blue area of the images of slightly moldy and moderately moldy wheat gradually became lighter, indicating that the degree of mold encapsulation on its surface gradually deepened. With the deepening of the degree of mold, the internal molds and various types of compounds in wheat gradually changed.

## 3 Identification of moldy wheat based on multi-scale context and feature pyramid

The raw THz images of samples acquired by the THz imaging system need to be further identified and classified by fine-grained classification algorithms. Aiming at the problems of deep learning-related traditional algorithms in the recognition process of moldy wheat, such as low recognition accuracy, we propose a moldy wheat recognition network based on multi-scale context and feature pyramid on the basis of ResNext.

### 3.1 Fine-grained classification algorithm MSCFP-Net for moldy wheat

**Figure 6:**
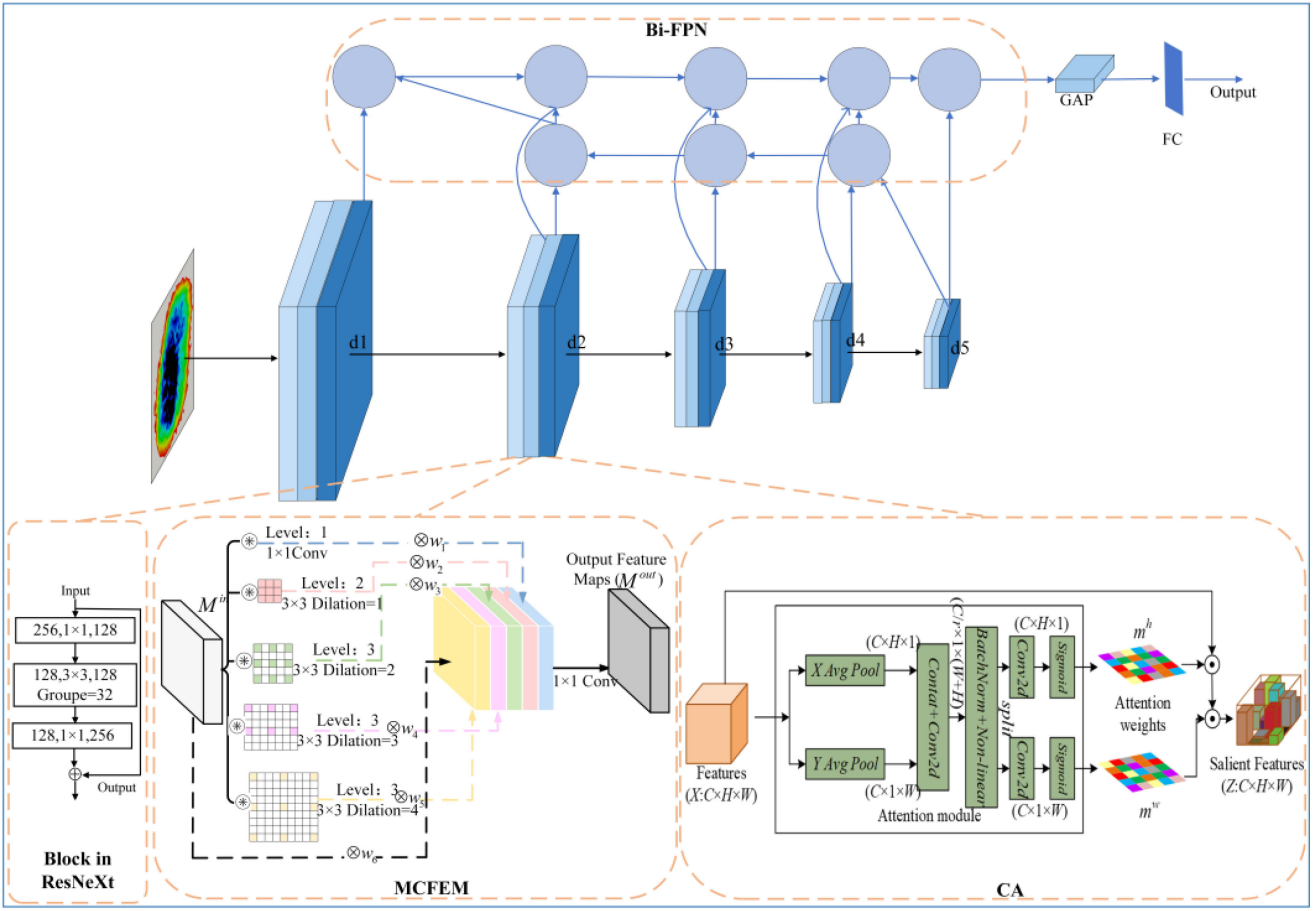
Overall structure of MSCFP-Net.

The overall network structure of the Multi-scale Context and Feature Pyramid based Network (MSCFP-Net) for moldy wheat recognition proposed in this chapter is shown in **Figure 6**. MSCFP-Net uses ResNeXt as the baseline model to extract deep complex features from samples with deeper network, thus achieving fine-grained image classification.

Based on ResNeXt, the first improvement module proposed is the multi-scale contextual feature extraction module. Since the extraction of contextual information helps to determine the important regions in the whole image so as to enable image detail feature extraction and improve the image recognition accuracy, and the fine-grained image classification task aims to achieve high-precision classification by obtaining the discriminative features of the important regions, this paper utilizes the idea of multi-scale contextual information to construct a feature extraction module and connects it with each block in the ResNeXt directly cascaded.

In addition, the THz image features of wheat with different moldy degrees have a very small gap, and there may be a situation where the network fails to focus on the discriminative features of different wheats, which may lead to misjudgment. The coordinated attention mechanism can guide the network to focus on the fine-grained discriminative features of the target region, and improve the ability of the network to learn the features by calculating the weights, so in this paper we cascade the multiscale contextual feature extraction module after each a coordinated attention module.

Moreover, in order to fuse the shallow feature maps enriched with more fine-grained features and the deep feature maps enriched with semantic features to obtain multi-scale fusion features and improve the recognition accuracy, this paper embeds a bidirectional pyramid feature extraction module in the baseline model and adopts cross-layer feature information connections to perform multi-scale feature fusion.

The input features are processed by five holistic modules consisting of a cascade of ResNeXt’s block branch, a multi-scale contextual feature extraction module, and a coordinated attention module, while the output of each module also serves as an input feature for each input channel of the bi-directional feature pyramid module, which is used to achieve feature fusion through the feature pyramid module. Finally, the output of the bi-directional feature pyramid module is then subjected to a global average pooling operation and a fully connected layer for feature dimensionality reduction and extraction to obtain the final output.

### 3.2 ResNeXt network architecture

**Figure 7:**
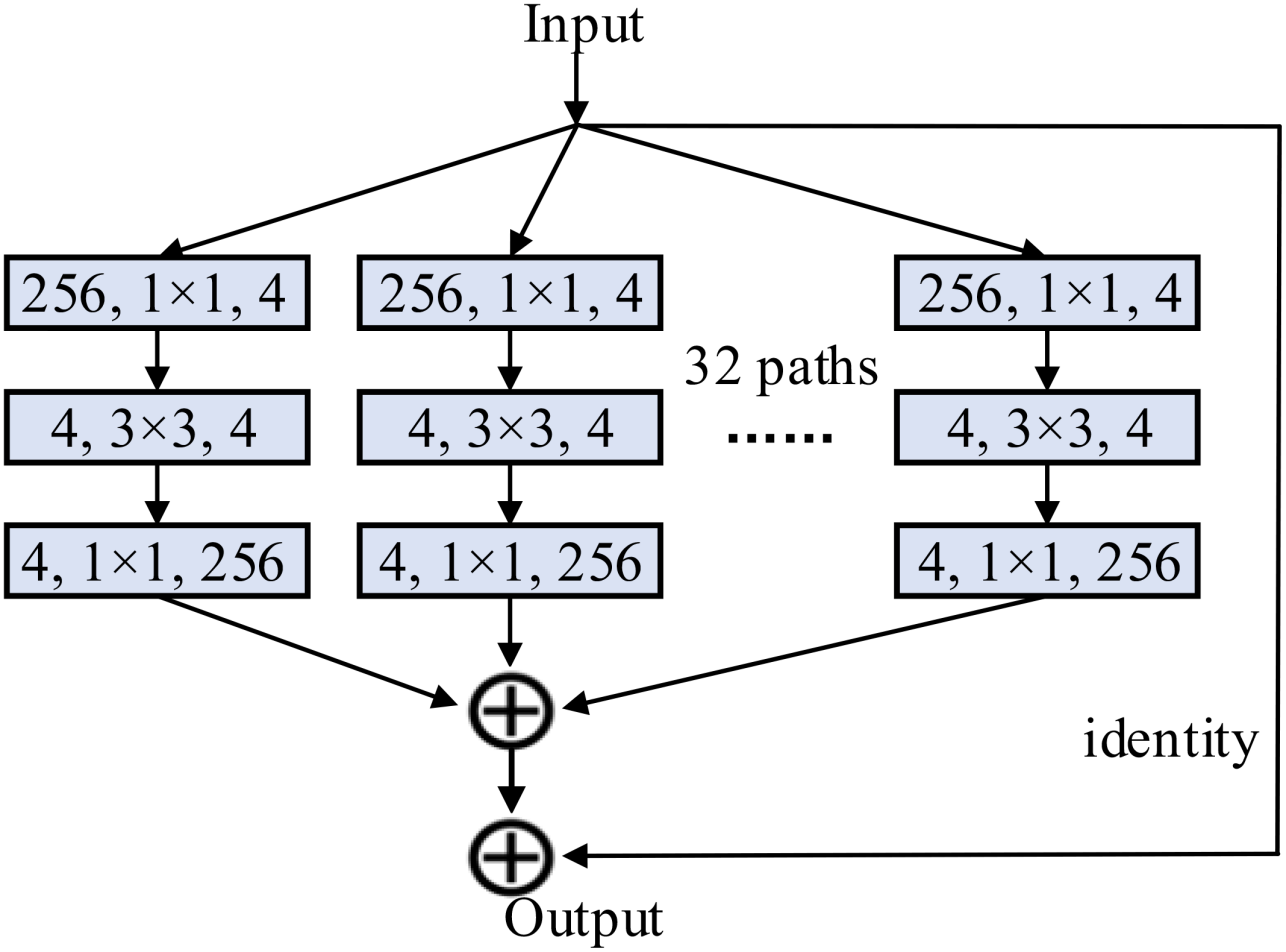
The structure of a block in ResNeXt.

Deep learning models make predictions closer to actual results by fitting an objective function, and numerous researchers are currently stacking CNNs to obtain more features. However, when the network layers are stacked up to a certain number, the problem of vanishing gradients occurs and the network performance degrades instead. The emergence of residual networks can effectively solve the gradient vanishing caused by excessive network stacking. The ResNeXt network (Xie et al., 2017) can be seen as a variant of the residual network, mainly replacing the previous residual structure with another block structure and using the concept of group convolution. It consists of multiple repeated blocks stacked together, where each block contains multiple identical parallel branches, and the results of each branch are summed and connected using residuals. The structure of each block is shown in **Figure 7**. For a feature map $F={\mathbb{R}}^{256\times H\times W}$ with 256 channels, it is first channel-compressed by 32 convolutional kernels of size 1×1 to generate a total of 32 sets of feature maps $F_{C}={\mathbb{R}}^{4\times H\times W},C=1,2,...,32$ with channel number 4. Then the size of the feature map is halved using convolution, and the 32 groups of feature maps $F_{C}={\mathbb{R}}^{4\times H\times W},C=1,2,...,32$ are channel upscaled by 32 convolutions with kernel size of 256 to generate 32 groups of feature maps $F_{N}={\mathbb{R}}^{256\times H\times W},N=1,2,...,32$ with 256 channels. Finally, the 32 sets of data are summed in the corresponding positions to synthesize a 256-channel output and a residual connection with the identity branch is formed, which ultimately constitutes the output of a block in ResNeXt.

**Figure 8:**
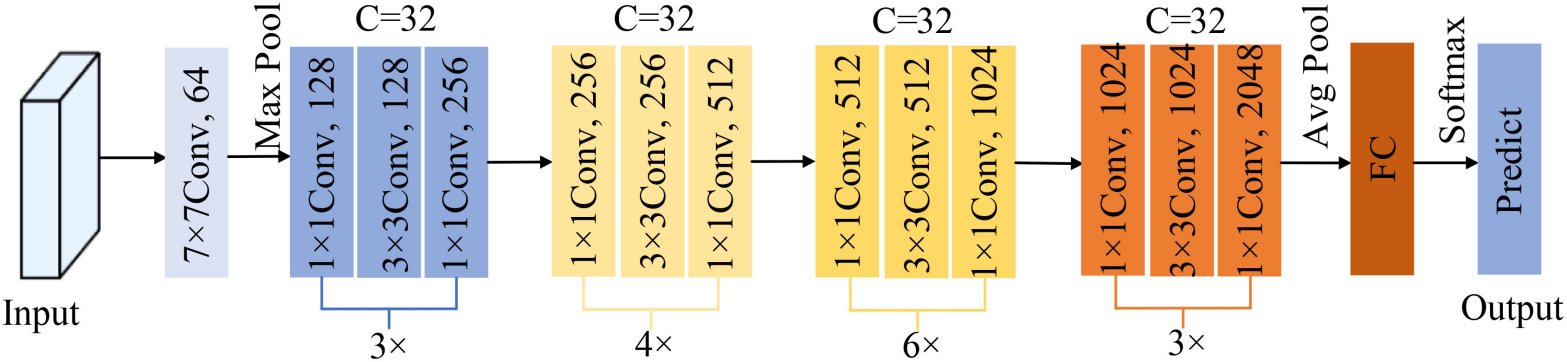
Structure of ResNeXt model.

The model structure of ResNeXt is shown in **Figure 8**, where C denotes the number of groups per block in the ResNeXt structure. As can be seen from the schematic diagram, it not only retains the residual idea of the ResNet model to avoid the gradient disappearance of the model caused by network stacking and deepens the network structure at the same time, but also adopts the parallel structure of the Inception module, which replaces the three-layer convolutional block of the original ResNet structure with the block composed of convolutional concatenated stacking, so that the model’s recognition accuracy is improved without increasing the parameter magnitude.

### 3.3 Multi-scale contextual feature extraction module

Traditional image classification networks, such as ResNet (He et al., 2016) and VGG (Simonyan and Zisserman, 2014), tend to focus only on increasing the depth to extract the deep semantic features of the image, which is effective for some image classification tasks with distinct differences in categorization features, such as cat and dog classification. However, in the fine-grained image classification task, high-precision classification can still not be achieved by deep semantic features alone, because for the traditional image classification tasks, the important discriminative features in an image, no matter how much weight they have, do not affect the network’s ability to extract the features in the same way for all regions of the image. For images where discriminative features occupy a small proportion, applying the same feature extraction method to all regions will result in the inclusion of a large amount of irrelevant background information along with important features in the network training, thereby affecting the model’s classification accuracy. Contextual information refers to the target and the relevant information features around the target area, and the extraction of contextual information helps to determine the important areas in the whole image. Traditional multi-scale feature extraction methods focus on independently extracting features from multiple scales. In contrast, the proposed multi-scale context and feature pyramid model integrates multi-scale feature extraction with contextual information, enabling the model to better capture multi-scale dependencies at different levels. This allows the model to extract more discriminative features from the most relevant regions. This approach reduces the interference of irrelevant background information on feature extraction, thereby improving the accuracy of image recognition.

**Figure 9:**
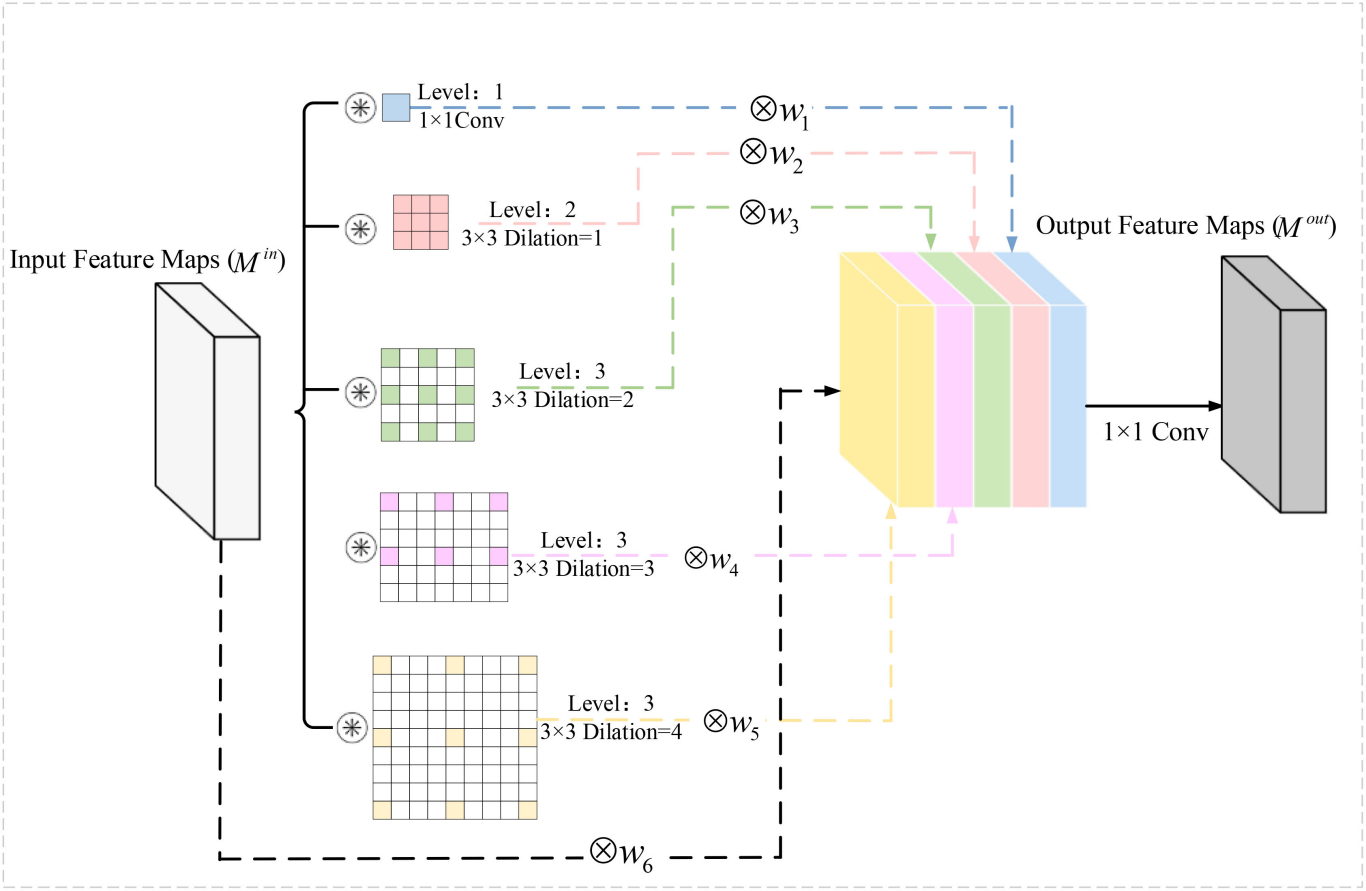
Contextual convolutional block structure.

Based on the above problems, a Multi-scale Context Feature Extraction Module (MCFEM) is proposed in this section, and the specific structure of MCFEM is shown in **Figure 9**.

MCFEM extracts contextual features at different scales by using a 1×1 convolution, a series of 3×3 convolutions with varying dilation rates, and its own feature mapping. Among them, the expansion rates of 3×3 convolution are 1, 2, 3, and 4. After a series of 3×3 null convolution and 1×1 convolution, the output feature map receptive fields are 1, 3, 5, 7, and 9, respectively. Small scale convolution helps in the extraction of detailed features in the feature maps, while large scale convolution facilitates the extraction of global features, and the introduction of null convolution achieves a certain degree of detail features retention while extracting different sensory field feature maps. The self-mapping of the residual structure is then introduced to form a total of six feature maps.

In addition, the idea of weighted fusion is used in MCFEM, and six learnable parameters *Z_i_*, *i* = 1,2,3,4,5,6 are provided in the module to enable the network to achieve adaptive learning of the importance of contextual features at different scales, where each weighting coefficient *W_i_* is determined by a learnable parameter *Z_i_*. *W_i_* is calculated as shown in Equation 1:


(1) $$W_{i}=\frac{e^{Z_{i}}}{\sum_{c=1}^{6}e^{Z_{c}}},i=1,2,3,...,6$$

The above approach allows the module to adaptively learn contextual features under different scales of sensory fields. Finally, the channels are downscaled using a 1×1 convolution to obtain the output features of the MCFEM.

In summary, MCFEM can merge contextual information from multiple scales and adaptively learn the importance of contextual features at different scales, where contextual information with small sensory fields is more beneficial for fine-grained classification tasks.

Considering that the convolution in the ResNeXt structure has scale singularity, and has limited ability to express the multi-scale features and detailed features of the moldy wheat image, a MCFEM is added before and after each block structure in the ResNeXt, so that each block has the ability to explore the contextual information of different scales, and then can extract the fine-grained features of the moldy wheat under different sensory fields.

### 3.4 Coordinated attention mechanism module

Compared to traditional image classification, fine-grained image classification needs to pay more attention to local salient features. The THz image features of wheat under different mold levels are very similar, so in order to avoid failing to pay attention to the discriminative features of different wheats, which may lead to misjudgment, a Coordinate Attention (CA) (Hou et al., 2021) module is added to each block of MSCFP-Net so that the network can pay more attention to the discriminative features in the THz images of wheat under different mold levels.

**Figure 10:**
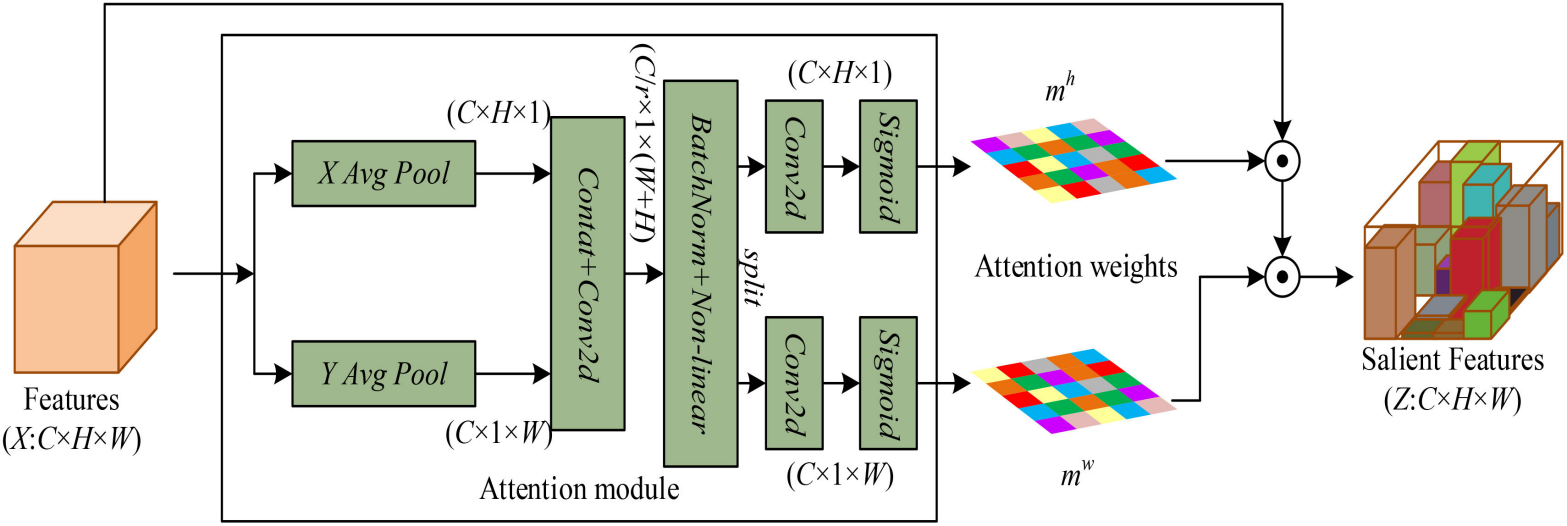
Structure of the coordinated attention mechanism module.

The structure of the CA module is shown in **Figure 10**. CA is an attentional mechanism used to enhance the network’s ability to learn feature representations and is designed to learn the importance of different regions in the input features by dynamically weighting them. The implementation of the CA module is divided into two parallel branches, horizontal and vertical.

A global average pooling operation is first performed for the input feature map $F\in {\mathbb{R}}^{C\times H\times W}$ from both horizontal and vertical directions to obtain the feature encoding mapping in both directions. The above process is achieved by using a pooling kernel of size (H,1) for global average pooling in the horizontal direction and using a pooling kernel of size (1,W) in the vertical direction. The final feature maps $y^{h}\in {\mathbb{R}}^{C\times H\times 1}$ and $y^{w}\in {\mathbb{R}}^{C\times 1\times W}$ after average pooling in both directions are obtained. The parallel pooling process for the cth channel feature output is shown in Equations 2 and 3.


(2) $$y_{\text{c}}^{h}(h)=\frac{1}{W}\sum_{0\leq i\leq W}x(h,i)$$

(3) $$y_{\text{c}}^{w}(w)=\frac{1}{H}\sum_{0\leq i\leq H}x(j,w)$$

After the above operation, the feature maps in both directions are then subjected to the concat operation, and then the nonlinear mapping of the features is carried out using the 1×1 convolution, the normalization layer and the activation function to generate the feature map $f\in {\mathbb{R}}^{\frac{C}{r}\times (H+W)}$ (*r* denoting the scaling factor). The above process can be expressed as Equation 4:


(4) $$f=\delta (F[y^{h},y^{w}])$$

The above approach allows the generated feature map $f\in {\mathbb{R}}^{\frac{C}{r}\times (H+W)}$ to fuse spatial information features in the horizontal and vertical directions, and then *f* to be decomposed along the spatial dimension to obtain $f\in {\mathbb{R}}^{\frac{C}{r}\times H}$ and $f\in {\mathbb{R}}^{\frac{C}{r}\times W}$ . Then it is subjected to channel expansion and nonlinear activation using 1×1 convolution and activation functions, respectively, to obtain the attentional weights in different directions of the spatial dimension $m^{h}\in {\mathbb{R}}^{C\times H\times 1}$ and $m^{w}\in {\mathbb{R}}^{C\times 1\times W}$ . Finally, the obtained attention weight values are multiplied with the input sequences respectively and modulated by the CA module to obtain the final output features.

In summary, a CA module is embedded in each block, so as to make the network pay more attention to the salient features of the target region.

### 3.5 Bi-FPN based feature fusion module

Traditional image classification networks usually use only the last layer of output as discriminative features for classification, and the deep features in the network are usually high-level semantic features. However, in fine-grained image classification tasks, the discriminative features of an image are often shallow detail information such as texture. Therefore, if only the output features of the last layer of the network are used as discriminative features, it may lead to poor classification accuracy. In this section, multi-scale feature fusion is used to fuse shallow feature maps enriched with more fine-grained features with deeper feature maps enriched with more semantic features, so that certain coarse-grained and fine-grained features are retained in the output features of the classification network at the same time. In this way, the accuracy of the network for fine-grained image classification tasks is improved.

With the advancement of deep learning, Feature pyramid is an important method in computer vision for handling multi-scale problems (Chen et al., 2024). Bi-directional Feature Pyramid Network (Bi-FPN) (Tan et al., 2020) is a bi-directional feature fusion model, which improves the recognition accuracy of target features through multi-level adaptive feature fusion and dynamic feature weight assignment. In addition, the model adopts cross-level feature information connection and multi-scale feature fusion to obtain more effective multi-level fusion features through the bidirectional cross-scale connection structure.

**Figure 11:**
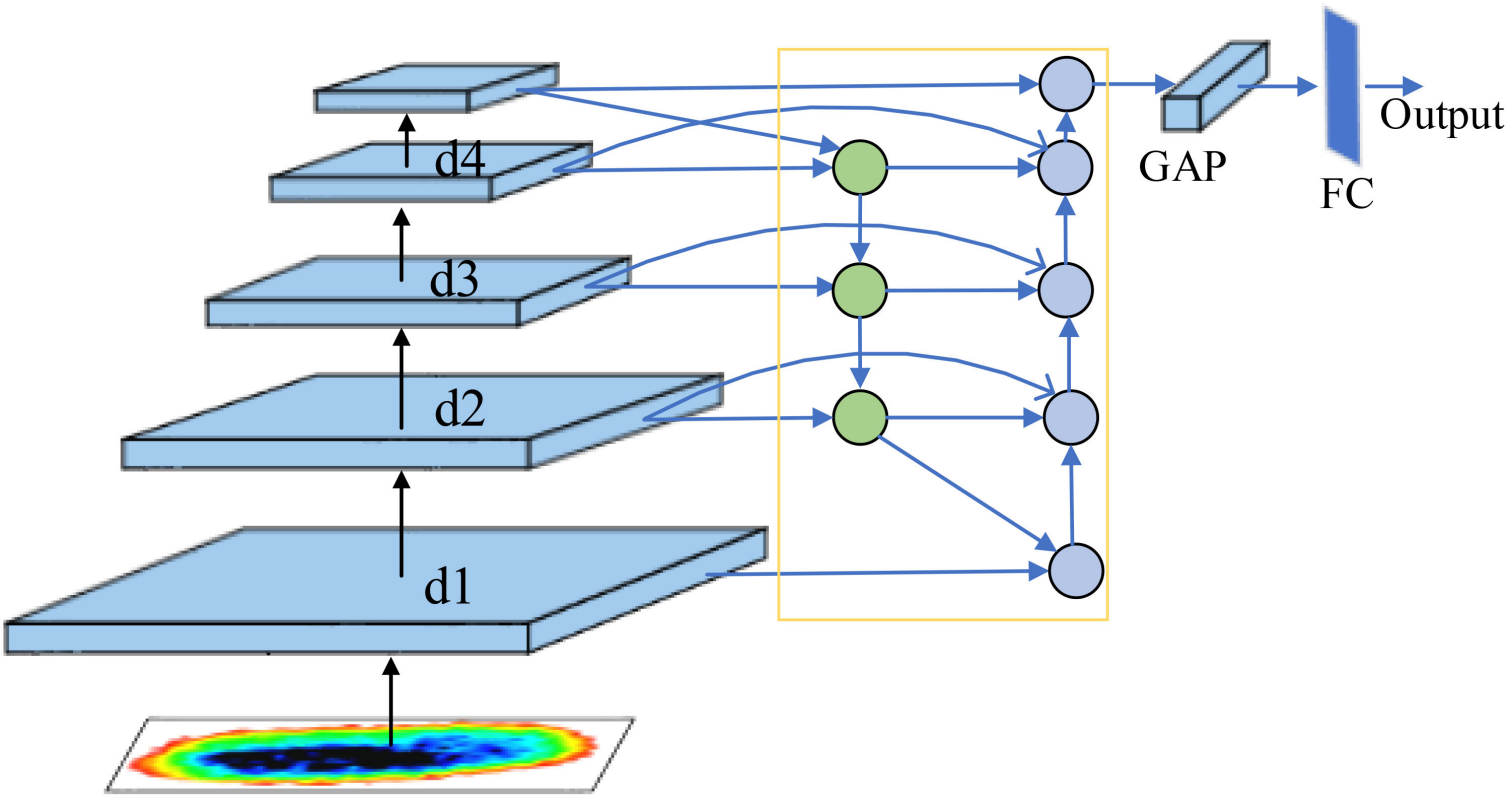
Network structure after entering Bi-FPN.

The specific connection of introducing Bi-FPN in ResNeXt is shown in **Figure 11**. The output feature maps of each block in ResNeXt are introduced into Bi-FPN, so that the shallow feature maps rich in fine-grained features are feature-fused with the deeper feature maps rich in semantic features, and the output features obtained from the topmost features of Bi-FPN are processed by global average pooling and fully-connected layers, respectively, for the classification task.

## 4 Experimental results and analysis

**Figure 12:**
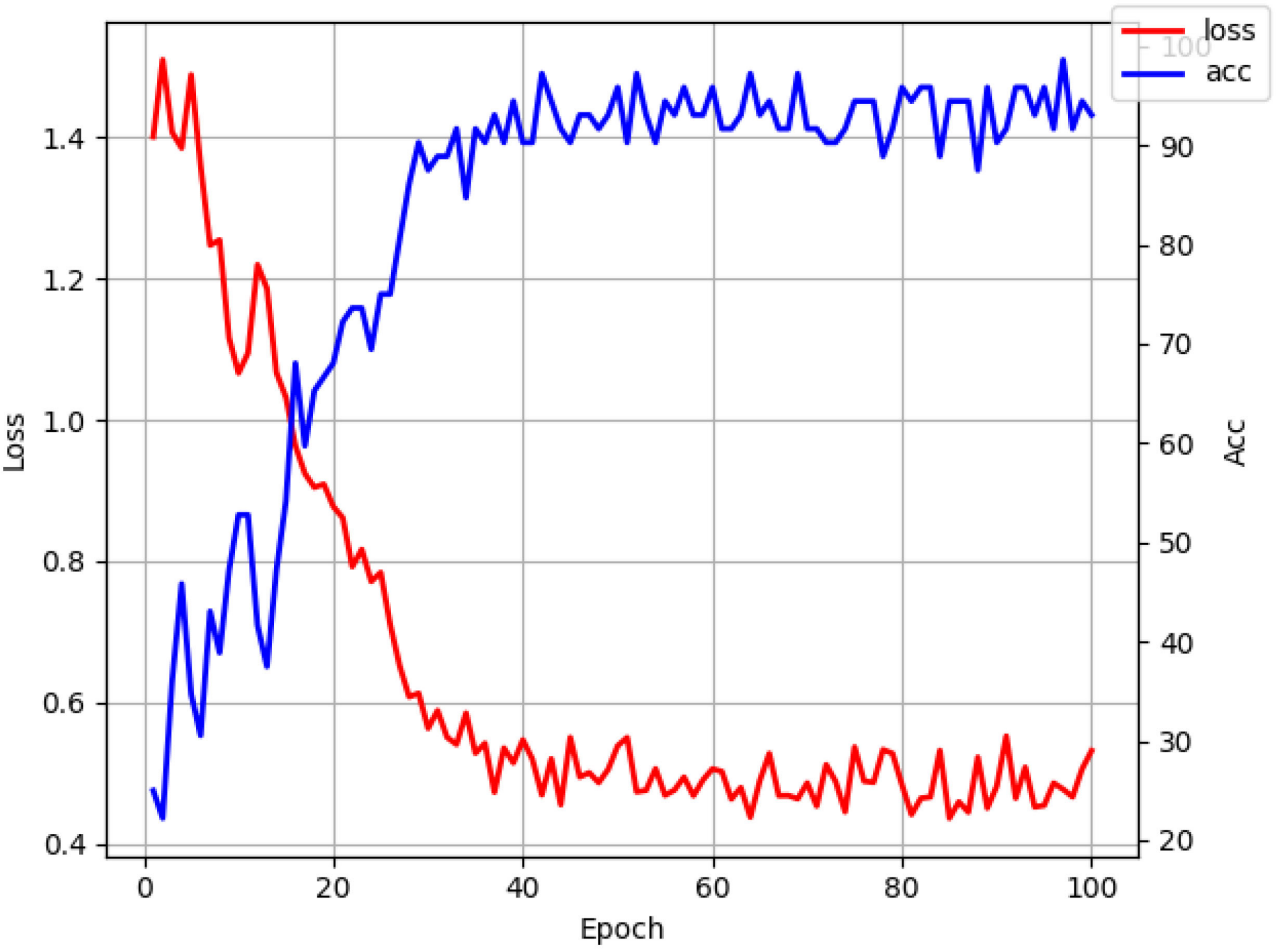
Loss function and accuracy change curves.

The 2D THz images of 1200 preprocessed wheat samples with different degrees of mold were divided into training and test sets in a 9:1 ratio, with 1080 and 120 images. The number of iterations for training is 100, using the SGD optimizer. The training set and labels used for model training were input into the MSCFP-Net constructed in this chapter for the classification process, and the loss function and accuracy changes of the model are shown in **Figure 12**. With the increase in the number of iterations, the model recognition accuracy increases rapidly, and when the epoch is close to 50 when the model training has stabilized, the accuracy growth slows down gradually and tends to 100%.

The specific values of the recognition results of the classification network in this paper are shown in **Table 1** As can be seen from the table, for the four samples the highest value of classification accuracy was 90.11%, while the lowest identification accuracy was found for mildly moldy wheat. In terms of recall value, 100% was achieved for both normal and seriously moldy wheat.

**Table 1:** Recognition results of MSCFP-Net (%).

Types	Normal	Slightly moldy	Moderately moldy	Seriously moldy
**Acc.**	87.50	85.71	**90.11**	88.56
**Rec.**	100	85.71	85.71	**100**
**F1.**	**92.33**	85.71	91.31	92.02

The bold values indicate the best results.

### 4.1 Model comparison

In order to further verify that the classification effect of the image recognition model constructed in this paper is better than the traditional recognition models, the mainstream classification networks DenseNet (Huang et al., 2017), ResNet, SENet (Hu et al., 2018), PVT-S (Wang et al., 2018), MobileNet-V2 (Howard et al., 2017), CSPDarkNet, ViT-B (Dosovitskiy et al., 2020) and ShuffleNet (Zhang et al., 2018) were chosen to class the THz images of moldy wheat and to compare and analyze the results of the above experiments with the results of the network in this paper from the point of view of the evaluation metrics Accuracy, Precision, Recall and F1-Score. The experimental results are shown in **Table 2**.

**Table 2:** Comparison of experimental results (%).

Networks	Acc.	Pre.	Rec.	F1.
DenseNet	84.65	82.47	86.87	84.61
ResNet	83.42	81.97	87.50	84.64
SENet	84.08	82.14	88.02	84.98
MobileNet-V2	85.06	82.98	88.73	85.67
CSPDarkNet	85.10	83.00	89.96	86.34
ShuffleNet	86.78	83.35	89.52	86.32
ViT-B	87.14	85.14	91.90	88.39
PVT-T	87.06	85.12	91.55	88.22
Baseline	86.89	85.02	91.73	88.25
MSCFP-Net	**87.97**	**86.27**	**92.26**	**89.16**

The bold values indicate the best results.

As can be seen from **Table 2**, the recognition performance of MSCFP-Net is optimal in terms of all the above four metrics. For the first eight mainstream networks, ViT-B and PVT-T reach the highest values of the four metrics, where the recognition accuracy is 0.25% and 0.17% higher than the benchmark networks in this paper, respectively. MSCFP-Net is 0.83%, 1.13%, 0.36% and 0.77% higher in the four metrics relative to ViT-B, which is the best performer among the top nine networks. In addition, MSCFP-Net has more performance improvement compared to the baseline network. Overall, the recognition accuracy and model stability of MSCFP-Net are optimal, indicating the superiority of the improvements of MSCFP-Net.

### 4.2 Ablation experiment

In order to verify that the MCFEM module, the CA module and the Bi-FPN module added to the baseline model in this chapter are all effective in improving the network recognition performance, ablation experiments are conducted on the dataset, and the performance comparison is made from the three perspectives of Accuracy, Recall, and F1-Score, respectively. The experimental results are the average values calculated for the four samples processed. As shown in **Table 3**, “√” indicates that the module is added, and “-” indicates that it is not added.

**Table 3:** Comparison of ablation experiment results (%).

Groups	MCFEM	CA	Bi-FPN module	ACC.	Pre.	Rec.	F1.
Group 1	–	–	–	86.89	85.02	91.73	88.25
Group 2	✓	–	–	86.97	85.12	91.98	88.42
Group 3	–	✓	–	86.95	85.16	91.82	88.36
Group 4	–	–	✓	86.98	85.13	92.00	88.43
Group 5	✓	✓	–	87.12	85.22	92.05	88.50
Group 6	✓	–	✓	87.56	85.96	92.21	88.98
Group 7	–	✓	✓	87.68	85.90	92.13	88.91
Group 8	✓	✓	✓	**87.97**	**86.27**	**92.26**	**89.16**

The bold values indicate the best results.

As can be seen from **Table 3**, among the eight groups of ablation experiments, the average recognition accuracy of the last group is the highest value, reaching 87.97%, which is significantly higher than that of the combined network with only one module added to the baseline network, and also higher than that of the combined network with two modules relative to those of the combined network with two modules, by 0.85%, 0.41% and 0.29%, respectively. The recognition accuracy in the last set of network is the only one that reaches more than 87%. Compared with the baseline network, the MSCFP-Net constructed in this paper improves the recognition accuracy of normal wheat by 1.08%. Experiments show that the MCFEM module, CA module and Bi-FPN module in the network proposed in this chapter effectively extract the multi-dimensional detailed features of the image and achieve effective feature fusion, which improves the recognition and classification ability of the samples, and can effectively perform the classification and recognition of moldy wheat.

From the comparison results of Accuracy, Precision, Recall and F1-Score, the average values of the metrics proposed in this paper are all the highest. After the experimental results and theoretical analysis, the performance of MSCFP-Net proposed in this paper is optimal in all four evaluation indexes. The network can effectively process THz image features of moldy wheat and accomplish high-precision classification.

### 4.3 Comparison of attention modules

The attention mechanism added to the MSCFP-Net is the CA module, so in order to prove the effectiveness of the CA module for the performance enhancement of this network, Selective Kernal (SK) (Li et al., 2019), Convolutional Block Attention Module (CBAM) (Woo et al., 2018), Efficient Channel Attention (ECA) (Wang et al., 2020) were embedded in MSCFP-Net respectively, with which the performance is compared. The comparison results are shown in **Table 4**.

**Table 4:** Comparison of recognition performance under different attention modules (%).

Model	Acc.	Pre.	Rec.	F1.
Baseline	86.89	85.02	91.73	88.25
+SK	86.92	85.88	92.08	88.87
+CBAM	86.26	86.14	92.14	89.04
+ECA	86.45	86.10	92.13	89.01
+CA	**87.97**	**86.27**	**92.26**	**89.16**

The bold values indicate the best results.

From the experimental results, it can be seen that the addition of all the above four attention modules improves the network performance. Among them, the results of adding SK module and CBAM module are very close to each other, with only 0.66%, 0.26%, 0.06% and 0.17% difference in the four metrics respectively. The recognition results of the network embedded with CA module in this paper reach the highest value, these four metrics higher than the experimental results of the network embedded with ECA module by 1.52%, 0.17%, 0.13%, and 0.15%, respectively. This is due to the fact that the coordinated attention mechanism enables simultaneous encoding of feature information in different dimensions and channels, directing the network to focus on fine-grained discriminative features in the target region and avoiding misjudgment of similar features.

Although the proposed model achieves a relatively high accuracy in identifying moldy wheat, there are still some limitations. In terms of model design, MSCFP-Net does not yet meet the requirements of high real-time performance in inference speed and computational efficiency, which restricts its potential for large-scale detection applications. In addition, the experimental dataset used in this study involves only a single wheat variety, and the generalization ability of the model needs to be further validated on more varieties and under diverse environmental conditions. With the rapid development of deep learning technologies, future work may focus on developing novel network architectures with higher accuracy and lower latency. Subsequent research can be carried out in the following directions: (1) optimizing the lightweight design of the network to improve real-time detection performance; (2) enhancing the model’s generalization ability through transfer learning; and (3) exploring multimodal data fusion strategies to further improve classification accuracy.

## 5 Conclusion

In this paper, a moldy wheat recognition network based on multi-scale context and feature pyramid is proposed around the high-precision recognition and fine-grained classification of moldy wheat, combining with the deep learning theory, thus realizing the high-precision classification of moldy wheat. The method uses ResNeXt as the baseline network, and firstly constructs a kind of multi-scale contextual feature extraction module to obtain the contextual semantic information of the target features and determine the important discriminative regions of the image; then the coordinated attention module is introduced to obtain more fine-grained features in the discriminative region; moreover, by embedding the Bi-FPN, the output feature information with the fusion of deep features and shallow features is obtained to improve the recognition accuracy. Extensive comparative experiments demonstrate that MSCFP-Net improves the recognition accuracy by 0.85% to 4.55%, significantly outperforming other benchmark networks in identifying moldy wheat using THz images. In addition, the model achieved a precision of 86.27% and a recall of 92.26%, enabling high-accuracy classification of wheat samples with varying degrees of mold contamination.

## Data Availability

The raw data supporting the conclusions of this article will be made available by the authors, without undue reservation.
